# Severe Emphysema Treated by Endoscopic Bronchial Volume Reduction with Lung Sealant (AeriSeal)

**DOI:** 10.1155/2013/361391

**Published:** 2013-04-23

**Authors:** R. F. Falkenstern-Ge, H. Ingerl, M. Kohlhäufl

**Affiliations:** Division of Pulmonology, Klinik Schillerhoehe, Center for Pulmonology and Thoracic Surgery, Teaching Hospital of the University of Tuebingen, Solitude Street 18, Gerlingen, 70839 Stuttgart, Germany

## Abstract

Endoscopic lung volume reduction using lung sealant is a very new and innovative treatment option for patients with severe progressive and irreversible lung emphysema. A 55-year-old ex-smoker (60 pack years) referred to our center because of severe lung emphysema with progressive worsening of the obstructive ventilator pattern and clinical condition. We detected collateral channels of this patient by using the Chartis system. Therefore, we decided to treat the advanced emphysema of our patient with endoscopic volume reduction using lung sealant (AeriSeal). The foam of lung sealant AeriSeal is instilled into the peripheral airways and alveoli where it polymerizes and functions as tissue glue on the lung surface in order to seal the target region to cause durable irreversible absorption atelectasis. The follow-up evaluation 12 weeks later showed improved lung function (increased FEV 1/partial oxygen pressure/peripheral oxygen saturation and a reduction of TLC and RV) with improved quality of life. Correlation between changes in primary and secondary outcome measures in the lung function parameters and 6-minute-walking test before and 12 weeks after the application of lung sealant revealed significant reduction of hyperinflation and improvement both in the flow rates and in the physical capability of this patient.

## 1. Case Presentation

**Figure 1:**
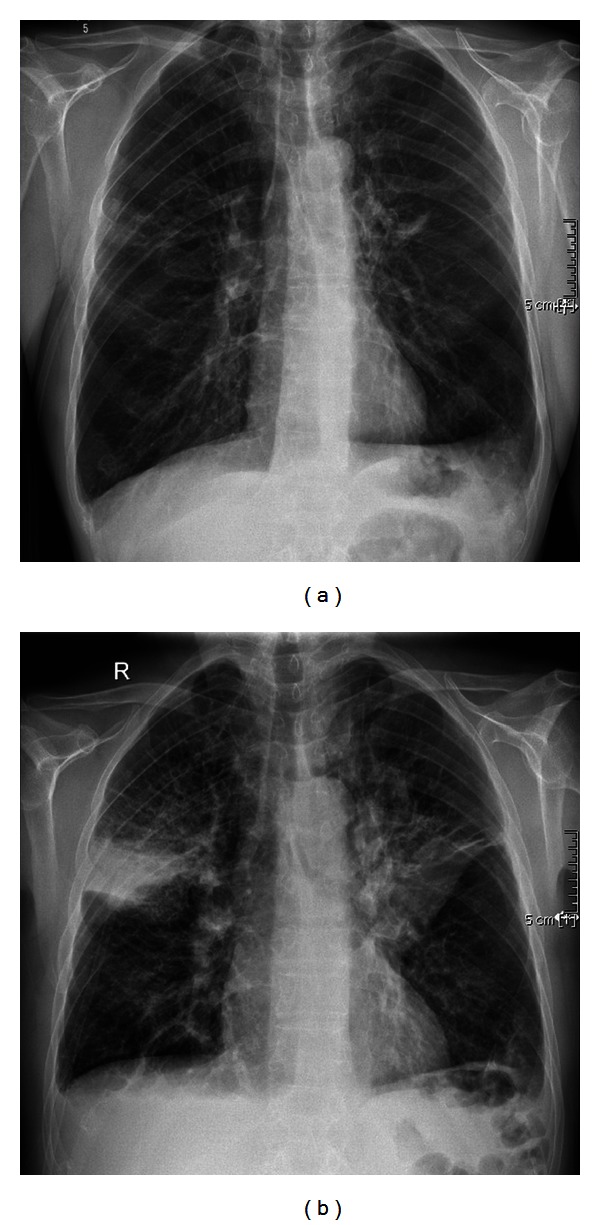
(a) Chest X-ray 4 weeks before the endoscopic lung volume reduction. (b) Chest X-ray 3 days after the endoscopic lung volume reduction shows atelectasis of both right and left upper lobe after endoscopic lung volume reduction.

**Figure 2:**
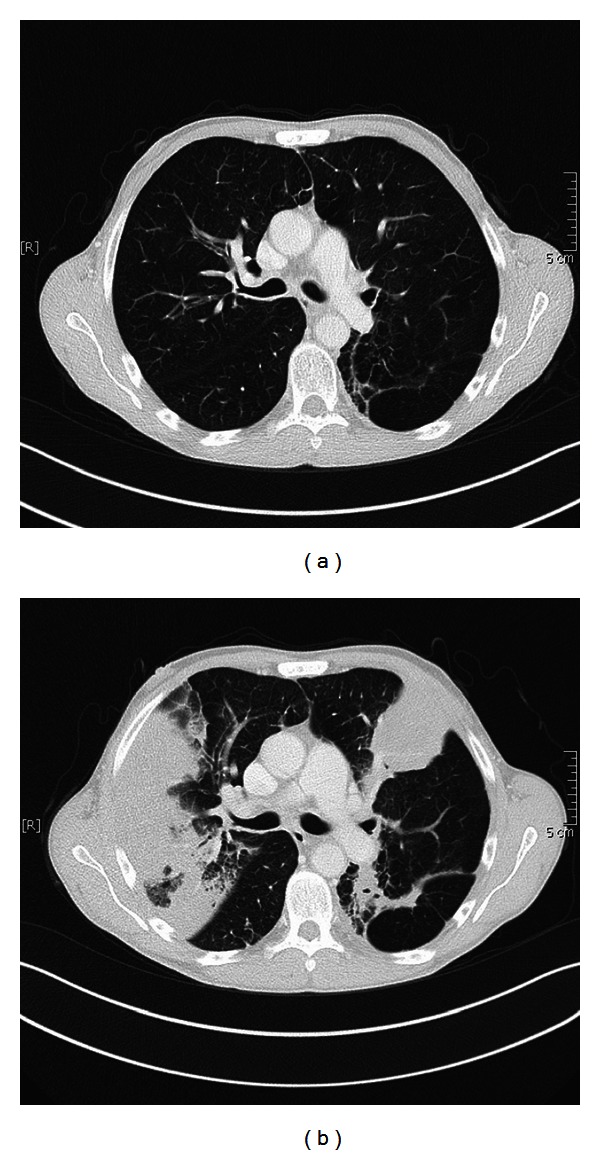
(a). CT scan 4 weeks before the intervention of endoscopic lung volume reduction revealed severe heterogeneous centrilobular lung emphysema with bilateral involvement of the upper lobes. (b) CT scan 3 days after the intervention of AeriSeal revealed the induced atelectasis of the bilateral upper lobes.

A 55-year-old ex-smoker (60 pack years) was referred to our center because of severe lung emphysema with progressive worsening of the obstructive ventilatory pattern and clinical condition despite maximized treatment and supplemental oxygen support. Due to limited treatment options, the therapy of endoscopic lung volume reduction was evaluated in our center. The chest X-ray (Figure 1(a)) and CT scan 4 weeks before the intervention with ELS revealed severe heterogeneous centrilobular lung emphysema with predominant involvement of bilateral upper lobes (Figure 2(a)). Collateral flow within the targeted area was proven by using the Chartis system; therefore, endobronchial valve treatment was not considered. Collateral channels can allow airflow into the target lobe and prevent atelectasis and significant lung volume reduction. We decided to treat the severe advanced emphysema of this patient with endoscopic volume reduction using lung sealant (AeriSeal). Follow-up evaluation with chest X-ray (Figure 1(b)) and with CT scan (Figure 2(b)) 3 days after the intervention showed visible atelectasis of both targeted upper lobes.

Our therapy was administered with the bronchoscope in correct wedge position at the airway subsegment. Synthetic polymer sealant foam was delivered through a single lumen catheter with its tip positioned 2 cm beyond the bronchoscope. Wedge position was maintained throughout delivery to prevent backflow into the airway.

**Figure 3:**
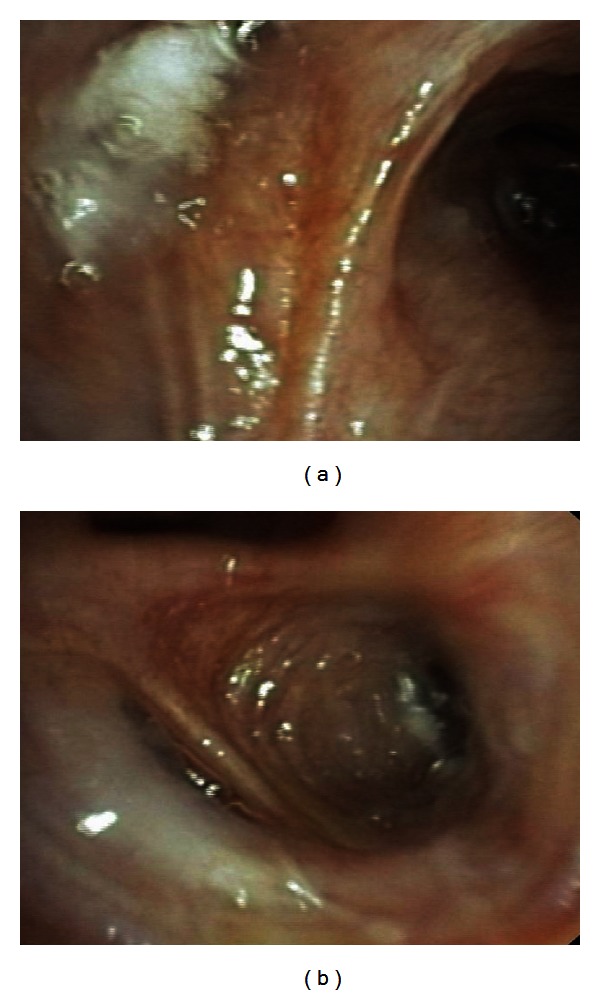
(a) AeriSeal foam sealants fill the targeted region of right upper lobe segment 2. (b) AeriSeal foam sealants fill the targeted region of left upper lobe segment 2.

The foam sealant was prepared at the bedside from aqueous polymer solution and cross-linker. Polymer solution contains 2% aminated polyvinyl alcohol in phosphate buffer. The 5 mL solution was mixed with 15 mL of air to generate 20 mL of foam sealant by passing the material back and forth through a stopcock between the syringes 10 times. 20 mL of liquid foam sealant was injected over 10–20 s. Wedge position was maintained for 1 min following delivery to allow complete in situ polymerization. The scope was then repositioned at the next treatment site, and the procedure repeated until all treatments were completed [1]. The bronchoscopic images (Figures 3(a) and 3(b)) showed that AeriSeal foam sealant fills the targeted diseased alveolar region right and left upper lobe segment 2, where it is intended to induce atelectasis and to block collateral air flow. After the instillation of the foam sealant, the patient had not experienced fever, leukocytosis, or increased sputum production.

Twelve weeks after the polymeric foam sealant application, the patient was clinically in a good condition, and reported improvement of general ability to manage daily life as well as an improvement of dyspnea. Pulmonary function test showed significant reduction of lung hyperinflation.

The parameter of the lung function examination also showed improvement of different parameters (Table 1).

## 2. Discussion

The following bronchoscopic lung volume reduction (BLVR) approaches have led to later-stage clinical trials: firstly, placement of endobronchial one-way valves designed to promote atelectasis by blocking inspiratory flow; secondly, formation of airway bypass tracts using a radiofrequency catheter designed to facilitate emptying of damaged lung regions with long expiratory times; thirdly, instillation of biological adhesives designed to collapse and remodel hyperinflated lung; fourthly, airway implants of nitinol coils of 10 to 20 cm in length designed for use in patients with either homogeneous or heterogeneous emphysema. These implants coil up on deployment and tether the lung.

Bronchoscopic lung volume reduction (BLVR) is a general term that refers to any of several recently developed endobronchial procedures for treating hyperinflation in advanced severe lung emphysema. Bronchoscopic lung volume reduction system with biological sealant/remodeling system is an alternative treatment option especially for patients with significant collateral flow which can easily be measured by the Chartis system. Like valve-based systems, it is designed to reduce lung volume directly by collapsing and sealing damaged areas of hyperinflated lung in patients with heterogeneous emphysema to reduce hyperinflation and to improve pulmonary function and quality of life in patients with advanced emphysema and collateral flow [2, 3]. Treatment by a biological sealant produces an irreversible change in emphysematous tissue. Biological sealant is delivered to the alveolar compartment as separate liquid components via a dual lumen catheter passed through the instrument channel of a flexible bronchoscope [4].

A common side effect is a systemic flu-like inflammatory reaction after foam sealant application accompanied by transient fever, cough, bronchospasm, chest pain, leukocytosis, malaise, and elevated C-reactive protein levels. This side effect is generally self-limited and resolves within 24–96 h spontaneously. Other serious pulmonary side effects within 6 months after the procedure include repetitive COPD exacerbations, pneumonia, bronchitis, and hemoptysis. Over a period of several weeks, the treated lung region will start to shrink, reducing lung volume by atelectasis [5].

Previous data from the National Emphysema Treatment Trial (NETT) showed that patients with heterogenous disease with low exercise capacity and both FEV_1_ and DL_CO_ > of more than 20% predicted could benefit from LVRS, demonstrating improvements in symptoms and physiology and reduced mortality [6, 7]. Lung volume reduction surgery (LVRS) reduces hyperinflation and improves lung function by removal of emphysematous lung tissue. However, LVRS is also associated with significant short-term morbidity and mortality [8].

Results from recently published Endobronchial Valve for Emphysema Palliation Trail (VENT) and Exhale Airway Stents for Emphysema (EASE) Trial showed that treatment was substantially less effective and did not consistently reduce hyperinflation or improve lung function mostly likely due to collateral ventilation present in majority of patients [9, 10]. The innovative therapeutic method with ELS is primarily considered for patients with detected collateral flow.

In our clinical documentation, the bronchoscopic lung volume reduction by a lung sealant showed significant improvement in both clinical status and lung function parameters. Future multicentral qualified studies are still necessary in order to identify the long-term effects of this innovative therapy.

## Figures and Tables

**Table 1:** Comparisons of the lung function parameters and 6-minute-walking test before and after endoscopic lung volume reduction.

Lung function parameter	Before application of AeriSeal	12 weeks after application of AeriSeal
Peripheral oxygen saturation with 2 L supply	93%	98%
Oxygen partial pressure	58 mmHg	89 mmHg
FEV 1 L (% pred)	1.3 (37)	1.4 (44)
Forced vital capacity (FVC) L (% pred)	3.2 (76)	3.2 (76)
TLCOc SB mmol/min/kPa	21%	25%
TLCOc/VA mmol/min/kPa	24%	28%
Total lung capacity (TLC) L (% pred)	7.9 (122%)	7.6 (117%)
Residual volume L (% pred)	4.7 (216)	4.5 (202)
SR tot (kPa·s) (% pred)	2.6 (217)	1.79 (151)
6-minute-walking test (m)	150	230
